# Characteristics of recurrent cases after conservative therapy in adolescent lumbar spondylolysis

**DOI:** 10.1038/s41598-022-07884-z

**Published:** 2022-03-07

**Authors:** Masaki Tatsumura, Hisanori Gamada, Shun Okuwaki, Fumihiko Eto, Katsuya Nagashima, Takeo Mammoto, Atsushi Hirano, Toru Funayama, Masashi Yamazaki

**Affiliations:** 1 Department of Orthopaedic Surgery and Sports Medicine, Tsukuba University Hospital Mito Clinical Education and Training Center/Mito Kyodo General Hospital, 3-2-7 Miyamachi, Mito, Ibaraki 310-0015 Japan; 2 Department of Orthopaedic Surgery, Faculty of Medicine, University of Tsukuba, Tsukuba, Japan

**Keywords:** Outcomes research, Paediatric research, Medical research

## Abstract

Occasionally lumbar spondylolysis in adolescents will recur after conservative treatment. The goal of this study was to retrospectively review the conditions in which recurrence transpired in a subset of adolescent patients diagnosed with acute lumbar spondylolysis. A retrospective survey was conducted in 141 patients who had been treated for spondylolysis and had obtained bone union. Twenty subjects were selected who had recurrent lumbar spondylolysis after returning to sports activity following the initial spondylolysis treatment. There were 18 males and two females with an average age at the time of initial visit of 13.3 years and 14.1 years at the time of recurrence. The average period of initial treatment was 101 days, and the average time to recurrence after healing was 149 days. There were three cases at L3, two cases at L4 and 15 cases at L5. At recurrence, 18 patients had unilateral involvement and two patients presented with bilateral occurrence. Four cases did not achieve bony union. In this study, the recurrence rate was 13.2%. Eighty percent of cases had recurrence within six months after healing. After recurrence, 20% of the cases reached pseudoarthrosis. It is useful to take regular MRI images to detect recurrence within six months after returning to sports.

## Introduction

Lumbar spondylolysis is a fatigue fracture of the pars interarticularis of the lumbar lamina and is known to occur frequently in young and adolescent athletes^1^. The initial symptoms often involve prolonged low back pain but early diagnosis has been made possible by the recent use of CT and MRI^2^. These imaging technologies have made it clear that lumbar spondylolysis sometimes can recur after healing. There are only a few reports on recurrence of lumbar spondylolysis. However, the frequency of recurrence has been reported to be 26%, which means that it is relatively common^3^. The recurrence rate may differ depending on the timing of return to sports activity. An early return to sports activity may cause recurrence of spondylolysis because the bone strength of the healed lesion is insufficient. We hypothesized that if the treatment period was appropriate, the recurrence rate could be controlled. The aim of this study was to identify in a retrospective study the conditions of repeated lumbar spondylolysis,

## Subjects and methods

Of 141 patients (male:112, female: 29) between 2014 and 2020 who were diagnosed with acute spondylolysis with bone marrow edema on magnetic resonance imaging (MRI) at the first visit and obtained bone union by conservative treatment, 22 lesions in 20 patients with recurrence at the same site after returning to sports activity were included in this study (recurrent group). Of these, two patients had two recurrences, but the analysis was limited to the first recurrence (2nd spondylolysis). Acute lumbar spondylolysis was defined as an MRI short tau inversion recovery (STIR) image showing a high intensity change around the pedicle. Conservative treatment was implemented by wearing a semi-hard brace and suspending sports activity. Termination of conservative treatment occurred when diminished bone marrow edema around the pedicle was observed by intensity normalization on MRI STIR. Bone union was evaluated by the presence or absence of bone continuity using computed tomography (CT) after verifying the disappearance of bone marrow edema using MRI^4^. All patients returned to the same sport in which they were engaged before injury after initial spondylolysis and healing. Recurrence was defined by bone marrow edema on the lesions that coincided with the once fused lesion. When the second spondylolysis occurred, conservative treatment was performed with a semi-hard brace and again suspending sports activity. Conservative treatment was continued until the disappearance of bone marrow edema. When intensity normalization was confirmed on MRI STIR, CT was performed. If CT showed continuity of the pars interarticularis, it was considered bony union. Lack of continuity of the pars interarticularis observed by CT was considered pseudoarthrosis. Pseudoarthrotic lesions that had no bone marrow edema on MRI at the first visit or the second visit were excluded.

We analyzed sex, age at the first visit, age at the recurrence, the treatment period during the initial spondylolysis, the treatment period during the recurrent spondylolysis, the period between initial healing and recurrence, vertebral level of the lesion, whether the lesion was unilateral or bilateral either at the first visit or at the recurrence, spina bifida occulta, CT staging at the first visit or recurrence, side (right or left) of the recurrent lesion, treatment result after recurrence, and sport discipline. We used 171 lesions in 121 cases who had healed once without recurrence as comparative controls for the analysis items (non-recurrent group).

The images were assessed independently by two orthopaedic surgeons who were unaware of the study participant’s status. Finally, a consensus decision was reached in conference. This research was approved by the IRB of Tsukuba University Hospital Mito Clinical Education and Training Center/ Mito Kyodo General Hospital, and the methods in this study were performed according to their guidelines and regulations. Written informed consent was obtained from the patients’ parents or legal guardians. The t-test was used for the age and treatment period and the significance level was set at 0.05.

### Ethics approval

All procedures including review of patient records used in this research were approved by the institutional review board.

### Consent to participate

 Written informed consents were obtained from all patients and their parent to participate in this study.

## Results

Eighteen males and two females with recurrence were included in recurrent group and 94 males and 27 females presented in the non-recurrent group. Table 1 shows Patient demographics. The average age at the first visit was 13.3 years (range: 10–17 years, standard deviation (SD): 1.92) and the average age at the time of recurrence was 14.1 years (range: 10–18 years, SD: 2.09) in the recurrent group. The average age at the first visit was 14.6 years (range: 8–18 years, SD: 1.93) in the non-recurrent group. The age of the recurrent group at the first visit was significantly lower than the age of the non-recurrent group at the first visit (P = 0.01). The average treatment period during the initial spondylolysis was 101 days (range: 31–213 days, SD: 42.1) and the average treatment period during the recurrent spondylolysis was 100 days (range: 36–182 days, SD: 44.8) in the recurrent group. The average treatment period was 104 days (range: 27–292 days, SD: 77.8) in the non-recurrent group. The treatment period of the recurrent group during the initial spondylolysis was not significantly different from the treatment period of the non-recurrent group (P > 0.05).

**Table 1:** Patient demographics.

		Recurrent group	Non-recurrent group
Number	Patients	20	121
	Lesions	22	171
Age at the first visit (years old)		13.3	14.6(※)
Age at the reccurence (years old)		14.1	
Treatment period at the first visit(days)		101	104
Treatment period at the reccurence(days)		100	
Vertebral level	L3 (lesions)	4	21
	L4 (lesions)	2	58
	L5 (lesions)	16	101
Laterality at the first visit	Unilateral (persons)	15	69
	Bilateral (persons)	5	52
Laterality at the reccurence	Unilateral (persons)	18	
	Bilateral (persons)	2	
Spina bifida occulta (persons)		15	59
Pathological stage at the first visit	Pre-lysis (lesions)	8	51
	Early (lesions)	14	96
	Progressive (lesions)	3	24
Pathological stage at the recurrence	Pre-lysis (lesions)	6	
	Early (lesions)	14	
	Progressive(lesions)	2	
Sport discipline	Baseball or softball (persons)	5	41
	Soccer (persons)	4	35
	Volleyball (persons)	2	13
	Track and field (persons)	2	6
	Swimming (persons)	2	2
	Others (persons)	5	24
			(※): *P* < 0.05

The number, age, treatment period, vertebral level of the lesion, laterality, spina bifida occulta, pathological stage, and sport discipline of recurrent group and non- recurrent group.

The period between healing and recurrence after healing was 149 days (range: 42–402 days, SD: 80.2). Sixteen patients presented with recurrence within six months after returning to sports activity.

The vertebral levels of the spondylolytic lesions in the recurrent group were L3 (4 lesions in 3 patients), L4 (2 lesions in 2 patients) and L5 (16 lesions in 15 patients) at the time of recurrence. The vertebral levels of the spondylolytic lesions in the non-recurrent group were L3 (21 lesions in 15 patients), L4 (58 lesions in 40 patients) and L5 (101 lesions in 72 patients). Six patients had spondylolytic lesions at the multiple vertebral body.

Fifteen patients had unilateral spondylolysis and five had bilateral spondylolysis at the first visit. Eighteen patients had unilateral and two had bilateral spondylolysis at the time of recurrence. Sixty-nine patients had unilateral and 52 patients had bilateral spondylolysis in the non-recurrent group. There were 15 patients with spina bifida occulta in the recurrent group and 59 patients with spina bifida occulta in the non-recurrent group. Thirteen unilateral cases had spina bifida occulta and the other five unilateral cases did not at the time of recurrence. Two bilateral cases had spina bifida occulta in the recurrent group.

Regarding CT staging, there were eight lesions in the pre-lysis stage, 14 lesions in the early stage, and three lesions in the progressive stage at the first visit and six lesions in the pre-lysis stage, 14 lesions in the early stage and two lesions in the progressive stage at the time of recurrence. In the non-recurrent group, there were 51 lesions in the pre-lysis stage, 96 lesions in the early stage and 24 lesions in the progressive stage.

At the time of recurrence, seven lesions were on the right side and 15 lesions were on the left side. Four patients did not achieve bony union.

All patients played a specific sport every day: In the recurrent group, five played baseball or softball; four played soccer; two each competed in volleyball, track and field, or swimming; and one each participated in karate, table tennis, tennis, badminton, or taekwondo. In the non-recurrent group, 41 played baseball or softball; 35 played soccer; 13 played volleyball; eight played basketball; six competed in track and field; five played tennis; two each participated in handball, kendo, wrestling or swimming; and one each participated in karate, judo, table tennis, field hockey, or figure skating.

## Illustrative case

**Figure 1:**
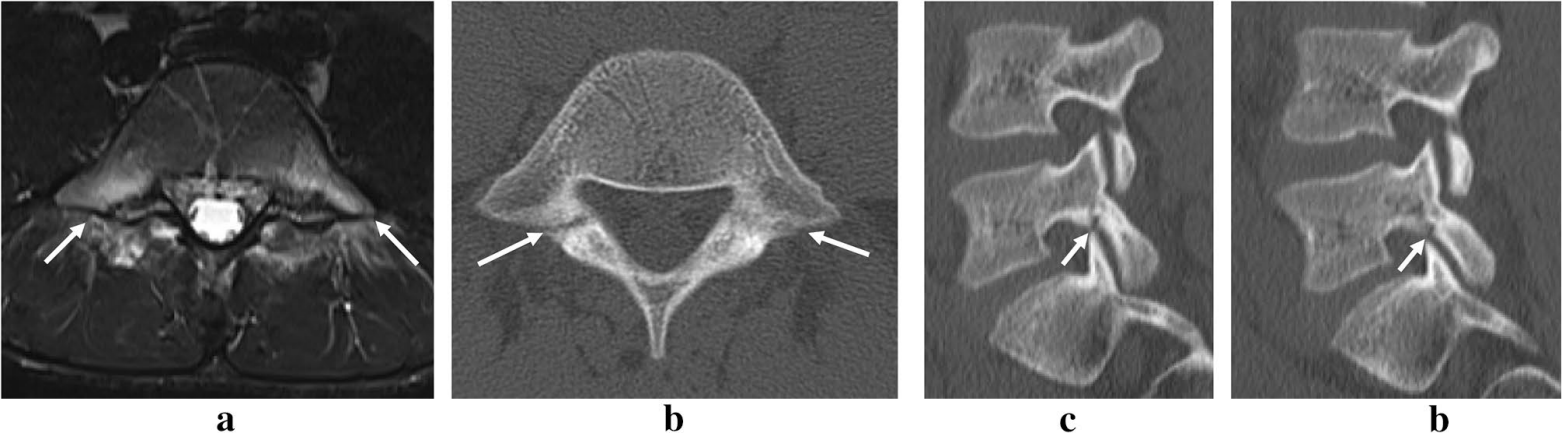
The short tau inversion recovery magnetic resonance images (STIR-MRI) at the first examination showed high intensity change bilaterally (white arrows) in the pedicles of L5 (**a**). CT revealed bilateral faint fracture lines (white arrows) on the axial slice (**b**) and slight bone resorption (white arrows) on the ventral side of the lamina in the sagittal view (**c**, **d**).

**Figure 2:**
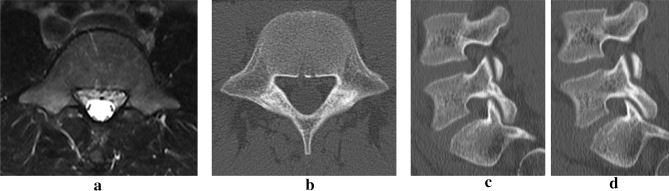
Nineteen weeks after the first examination, the intensity change on MRI disappeared (**a**). The fracture lines disappeared on the axial CT slice (**b**) and there was no obvious worsening of the resorption in the sagittal view (**c**, **d**).

**Figure 3:**
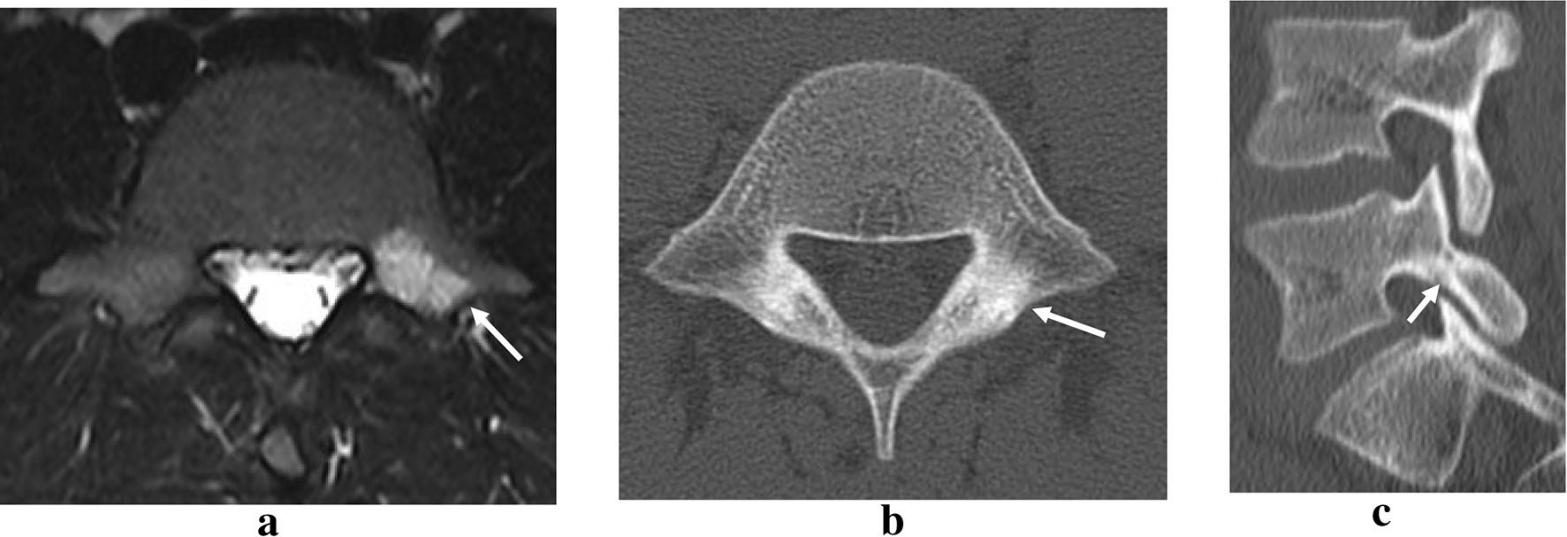
Twelve weeks after the first healing, short tau inversion recovery magnetic resonance images (STIR-MRI) showed high intensity change (white arrow) on only the left side (**a**). CT revealed no fracture line (white arrow) in the axial view (**b**) but slight bone resorption (white arrow) on the ventral side in the sagittal view (**c**).

**Figure 4:**
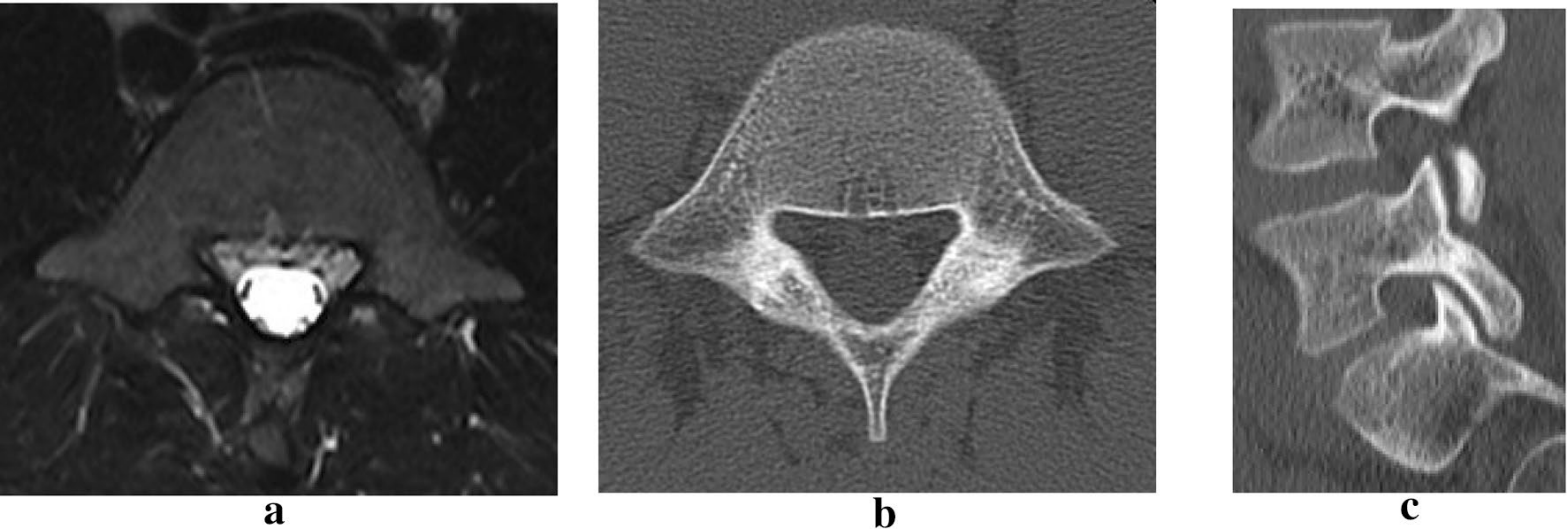
Ten weeks after the second conservative treatment, the intensity change on MRI disappeared (**a**). No abnormality was detected from the axial or sagittal CT slices (**b**, **c**).

A 14-year-old boy felt bilateral low back pain when he smashed a ball as a tennis player and presented to our hospital six weeks later. Physical examination revealed low back pain during lumbar extension and restricted range of motion in extension. At the first visit, the MRI STIR showed bone marrow edema on both sides of L5 (Fig. 1a). An axial view CT slice revealed fracture lines on both sides, which were classified as progressive on the right side and early stage on the left side (Fig. 1b). In the sagittal view, they were classified as 1a on the bilateral side (Fig. 1c, d). After conservative treatment, suspending sports activities, and a semi-hard brace for 19 weeks, bone healing was achieved with normal bone intensity on MRI (Fig. 2a) and bone continuity shown by CT (Fig. 2b-d). However, 12 weeks after healing and a return to playing tennis, bilateral L5 spondylolysis recurred. MRI showed bone marrow edema on the left side again (Fig. 3a). The spondylolysis was classified as pre-lysis stage on the left side from the axial MRI view (Fig. 3b) and as 1a on the left side from the sagittal view (Fig. 3c). Disappearing bone marrow edema and bony union were achieved with 10 weeks of the 2^nd^ round of conservative treatment (Fig. 4a-c) and he subsequently returned to tennis.

## Discussion

Treatment of lumbar spondylolysis causes athletes to leave competition for at least a few months. All patients in this study returned to their original sport after the initial healing of lumbar spondylolysis but returning to sports too early caused recurrence. From this survey, the period to recurrence was within six months after returning to sports in 16 of 20 patients.

Appropriate time of the return to sports activity may decrease spondylolysis because bone strength of the healed lesion is sufficient. In this study, the average period after healing until recurrence was 149 days; the recurrence of spondylolysis did not occur immediately after returning to exercise. Suspending sports activity for longer can reduce the recurrence rate but lengthening the time to return to sports is not desirable for athletes. Therefore, strength of the lesion was sufficient and the timing to resume sports was appropriate. The average treatment period for the initial spondylolysis was 101 days, and the treatment period after the recurrence was 100 days. There was no significant difference in the period of treatment between the recurrent and non-recurrent groups. Therefore, we conclude that the cause of the recurrence was not that the treatment period was too short. Even if the duration of treatment is appropriate, the recurrence will occur. All 20 patients returned to sports after the 2nd conservative treatment but two patients returned with a 3rd spondylolysis (2nd recurrence).

The age at first visit in the recurrent group was significantly lower than that in the non-recurrent group. Because adolescents are not physically mature, it is difficult to prevent 100% recurrence of lumbar spondylolysis in this age group, but that the patient had only partially recovered bone strength and the spondylolysis unfortunately recurred. The most of patients in both the recurrent and non-recurrent groups were visited within 4 weeks of the onset of pain, and the duration of symptoms before diagnosis was not considered to be different.

In the recurrent group, there were only two cases in the progressive stage which was complete lysis. As we suspected recurrence when pain appeared and advised patients to seek medical attention as early as possible, most patients came back to the hospital before complete lysis.

Recurrence in the earliest case occurred 42 days after healing. Therefore, recurrence may occur after an early return to sports. It will be necessary to instruct the athletes and parents to refrain from sudden high-intensity exercise immediately after resuming sports. On the other hand, there was a case of recurrence more than one year after the initial treatment. The possibility of recurrence of lumbar spondylolysis is not zero if the athlete continues to play competitive sports.

The rate of males in the non-recurrent group constituted 94/121 (77.7%) in this study. The rate of male in the recurrent group was 2/20(90%). The recurrence rate for males was 18/112 (16.1%) and for females was 2/29 (6.9%). Although the number of patients in this study was small, recurrence may be more likely to affect males.

There were five cases of bilateral spondylolysis at the first visit and two cases after recurrence. Once suffering from lumbar spondylolysis, the patient learns related symptoms such as the location of pain. As a result, because the patient tends to visit the hospital earlier and more cases can be diagnosed as unilateral before reaching bilaterality, the number of bilateral cases at the time of recurrence is lower. Therefore, post-treatment follow up is necessary given the possibility of recurrence and patients and their parents should be informed about the possibility of recurrence.

Non-union occurred in four cases after recurrence. One was bilateral, one was unilateral but at a progressive stage and the other two were unilateral incomplete fractures. Bilateral lesions are considered to be difficult to heal^5^, and union in those in a progressive stage also are considered to be difficult^4^.

Both the age of patients at the initial treatment and the recurrence, and the predominance of L5 in most cases of spondylolysis were consistent with previous reports^6^.

Even in cases in which union was obtained once, some patients had non-union after recurrence. It has been proposed that there is a predisposition for lumbar spondylolysis in some individuals^7^, and it is possible that the predisposition also affects the union rate. Individual characteristics may influence both fusion and the tendency for lumbar spondylolysis. As all recurrent cases had a history of bony fusion once, they have some potential to regenerate bone. However, this study showed that patients may not always heal even if the same treatment led to bony union in the past. We are paying close attention to recurrence after returning to exercise and are considering ways to detect recurrence earlier. As MRI is useful for early detection of lumbar spondylolysis^2^, it is important to regularly perform additional MRI analyses to confirm the presence or absence of recurrence. In addition, when low back pain appears after returning to sports following treatment for lumbar spondylolysis, MRIs should be taken early for diagnostic purposes.

The recurrence and non-recurrence groups were similar in age, CT staging, level, unilateral or bilateral, and sports discipline. No risk factors for recurrence could be identified among these factors.

The limitation of this study is that the number of cases is small and statistical examination is insufficient for univariate or multivariate analysis.

## Conclusion

In this study, the time to recurrence after healing averaged five months after return to sports activity. Not all patients obtained bony union after recurrence, even if they had achieved union after the initial treatment and healing period. As in prior studies, the most common vertebral level for spondylolysis was L5. Because recurrence can happen, it is useful to take regular MRI images even after returning to sports activity to detect recurrence at an early stage, especially if the patient is reporting lumbar pain.

## Data Availability

The datasets generated and analyzed during the current study are available from the corresponding author on reasonable request.
